# Injectable hyaluronic acid–metformin conjugate gel for sustained intra‐articular delivery and prevention of post‐traumatic osteoarthritis

**DOI:** 10.1002/btm2.70100

**Published:** 2025-12-25

**Authors:** Vasyl Pastukh, Jianying Zhang, Soichi Hattori, Susheng Tan, Satyaj Bhargava, Derek Maloney, MaCalus V. Hogan, James H‐C. Wang

**Affiliations:** ^1^ MechanoBiology Laboratory, Bethel Musculoskeletal Research Center, Department of Orthopaedic Surgery University of Pittsburgh School of Medicine Pittsburgh Pennsylvania USA; ^2^ Department of Electrical and Computer Engineering University of Pittsburgh Pittsburgh Pennsylvania USA; ^3^ Department of Bioengineering University of Pittsburgh Pittsburgh Pennsylvania USA; ^4^ Department of Physical Medicine and Rehabilitation University of Pittsburgh Pittsburgh Pennsylvania USA

**Keywords:** controlled drug delivery, hyaluronic acid–metformin conjugate, in vivo therapeutic evaluation, injectable hydrogels, post‐traumatic osteoarthritis

## Abstract

We developed an injectable hyaluronic acid–metformin (HA–Met) conjugate gel for localized intra‐articular delivery to mitigate post‐traumatic osteoarthritis (PTOA). Conjugation was verified by Fourier‐transform infrared spectroscopy, proton nuclear magnetic resonance spectroscopy, and high‐performance liquid chromatography. In vitro studies showed an initial 24‐h burst attributable to unbound Met, followed by prolonged local retention from the conjugated fraction under physiological incubation without hyaluronidase treatment; by contrast, a simple HA + Met mixture released Met in phosphate‐buffered saline (PBS) completely in 3 days. In high dosage hyaluronidase‐containing PBS, Met retained in the HA–Met conjugate gel up to 4 days. In vivo, Met remained detectable in mouse knee tissues for up to 7 days after a single intra‐articular injection of HA–Met conjugate and accumulated with weekly dosing; in contrast, after Met solution or HA + Met mixture injection around 99% of Met was removed from the knee joint in 1 day, low dosage traces of Met remained in the joint up to 2–3 days. In a destabilization of medial meniscus‐induced murine PTOA model, weekly HA–Met conjugate injections attenuated cartilage degeneration and joint pathology versus HA or Met alone, which produced only modest effects; sham joints exhibited no pathological changes. The weekly interval was selected to ensure continuous exposure in mice with rapid metabolism and joint clearance and should be viewed as an accelerated proof‐of‐concept schedule rather than a clinical regimen. These findings support HA–Met conjugate gel as a translational platform that achieves prolonged intra‐articular Met retention and disease‐modifying benefit in a small‐animal PTOA model.

## Graphical abstract

An injectable hyaluronic acid (HA)–metformin (Met) conjugate gel enables sustained intra‐articular retention of Met and significantly reduces cartilage degeneration in a murine post‐traumatic osteoarthritis (PTOA) model, highlighting a disease‐modifying strategy to prevent joint degeneration following injury.

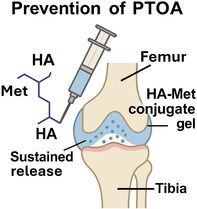

**Translational Impact Statements**

An injectable hyaluronic acid–metformin (HA–Met) conjugate gel was designed to provide localized delivery and prolonged intra‐articular retention of Met in injured joints. By covalently linking Met to HA, the gel harnesses its anti‐inflammatory, antioxidant, and chondroprotective activities to help prevent post‐traumatic osteoarthritis (PTOA). This approach addresses key limitations of current therapies by improving Met retention and reducing systemic exposure through covalent bonding rather than physical encapsulation. In a murine PTOA model, the conjugate preserved cartilage and maintained joint integrity when administered early after injury. Its scalable synthesis, established biocompatibility, and constituent safety record support its potential as a clinically translatable preventive therapy for post‐injury joint degeneration.

## 1 INTRODUCTION

Hyaluronic acid (HA) is a naturally occurring, biocompatible polysaccharide abundantly found in the extracellular matrix (ECM) of soft connective tissues, including articular cartilage and synovial fluid. Clinically, HA is approved for intra‐articular injection in mild osteoarthritis (OA) to restore lubrication, reduce friction, and improve joint mobility.^1^, ^2^, ^3^, ^4^, ^5^ Owing to its viscoelasticity, biodegradability, and tissue‐specific interactions, HA also serves as a versatile platform for intra‐articular drug delivery. However, when administered alone, HA provides only transient symptom relief and does not significantly alter the course of OA progression.^6^, ^7^

To overcome these limitations, HA‐based delivery systems have been engineered to enable sustained, localized release of therapeutic agents directly within injured joints. By increasing intra‐articular drug retention while minimizing systemic exposure, these systems aim to enhance therapeutic efficacy and duration. Among various strategies, chemical modification of HA—through covalent conjugation^8^ or encapsulation of bioactive molecules^9^—has shown promise for achieving targeted and bio‐responsive delivery within the joint microenvironment.

Metformin (Met), a widely used antidiabetic agent, has recently gained attention for its therapeutic potential in musculoskeletal conditions, including OA. OA is a degenerative joint disease with pathological features including cartilage damage, bone fragmentation, osteophytes, and synovitis. Recent studies have shown that AMP‐activated protein kinase (AMPK) is a central regulator of cellular metabolism, and activation of AMPK not only decreases inflammation but also inhibits the transmission of pain signals.^10^ Met, as an AMPK activator, has shown anti‐inflammatory capacity in an OA rat model.^11^

Pain is a common clinical manifestation of OA. An OA model study revealed that Met treatment decreased dorsal root ganglion pain in animals' sensitivity.^12^ More recent studies have demonstrated the analgesic effect of Met.^13^, ^14^ In preclinical studies, Met exhibits anti‐inflammatory, anti‐fibrotic, and chondroprotective properties, and has been shown to attenuate cartilage degradation, suppress fibroblast activation, and modulate nociceptive signaling pathways.^14^, ^15^, ^16^, ^17^, ^18^ Nevertheless, systemic administration of Met is hampered by rapid clearance, off‐target distribution, and the need for frequent dosing due to its short plasma half‐life (5–6 h).^19^, ^20^

To facilitate the stable and sustained release of Met at the site of the wounded tissue, many biodegradable hydrogel scaffolds were used as a carrier for Met delivery in the treatment of wounded tissue regeneration and repair. A Met‐loaded polycaprolactone (PCL) and polyvinyl alcohol (PVA) fibrous scaffold has been used to enhance bone regeneration.^21^ A Met‐loaded gelatin‐methacrylate hydrogel as a localized delivery approach has shown significant potential for enhancing bone regeneration.^22^

In this study, we developed a novel HA–Met conjugate gel designed for intra‐articular injection to provide localized, sustained delivery of Met to injured joints. By covalently linking Met to HA, the formulation integrates the structural and lubricating benefits of HA with the anti‐inflammatory activity of Met, resulting in a dual‐functional therapeutic platform. This chemical conjugation enhances drug retention within the joint, improves local bioavailability, and reduces systemic exposure.

To assess the therapeutic efficacy of the HA–Met conjugate, we employed a surgically induced destabilization of medial meniscus (DMM) model in mice, which recapitulates key pathological features of post‐traumatic osteoarthritis (PTOA). PTOA is a common and debilitating consequence of joint injury, particularly in younger and physically active individuals, yet current treatment options remain largely palliative. By targeting the early pathological processes following joint trauma, the HA–Met conjugate gel offers a disease‐modifying strategy with strong translational potential for preventing or delaying PTOA progression.

## 2 MATERIALS AND METHODS

### 2.1 Materials

Metformin hydrochloride (Cat. #PHR1084, pharmaceutical secondary standard, Sigma Aldrich), sodium hyaluronate (≥95% purity; CAS 9067‐32‐7, Acros Organics), 1‐ethyl‐3‐(dimethylaminopropyl) carbamide HCL (EDC) (CAS 25952‐53‐8, Thermo Scientific), *N*‐hydroxy succinimide (NHS) (≥ 98% purity, CAS 6066‐82‐6, Aldrich) were used for HA–Met conjugate synthesis. All the other chemicals and reagents used in this study were analytical grade.

### 2.2 Preparation of novel HA–Met conjugate hydrogel

The HA–Met conjugate hydrogel was synthesized as outlined in Figure S1. Sodium hyaluronate (200 mg, 1500 kDa) was dissolved in 20 mL of distilled water (10 mg/mL), followed by the addition of EDC (10 mg) and NHS (12.5 mg). The mixture was stirred for 30 min, then metformin hydrochloride (800 mg; 40 mg/mL final concentration) was added and stirred overnight at room temperature. The next day, 20 mL of acetone was added, and the resulting white solid was collected by centrifugation (2000 rpm, 10 min), washed with 2 mL of deionized water, and dried at 50°C overnight. The dried conjugate was re‐dissolved in deionized water to prepare the HA–Met conjugate hydrogel for further use.

### 2.3 HA–Met conjugate Fourier‐transform infrared spectroscopy

The conjugation of HA and Met was evaluated using Fourier‐transform infrared (FT‐IR) spectroscopy (Bruker VERTEX‐70LS), as previously described.^23^ The FT‐IR spectrum of HA was obtained following a modified protocol.^24^ The spectrum of Met was recorded according to a previously reported method.^25^ For each measurement, 4 mg of the solid sample was thoroughly mixed with 196 mg of IR‐grade potassium bromide (KBr), and the mixture was scanned over the wavenumber range of 4000–400 cm^−1^ using the FT‐IR spectrophotometer (Bruker).

### 2.4 HA–Met conjugate nuclear magnetic resonance spectroscopy

To confirm the chemical conjugation between HA and Met, proton nuclear magnetic resonance (^1^H NMR) spectroscopy was performed using a Bruker Avance III 500 MHz NMR spectrometer (Bruker BioSpin, Billerica, MA, USA).^26^ Briefly, HA, Met, and the HA–Met conjugate were each dissolved in deuterium oxide (D_2_O) at a concentration of approximately 10 mg/mL and transferred to 5 mm NMR tubes. Spectra were acquired at 22°C with 64 scans and a relaxation delay of 2 s. The resulting spectra were analyzed to identify characteristic peaks of HA and Met and to verify successful amide‐bond formation in the HA–Met conjugate.

### 2.5 High‐performance liquid chromatography analysis for HA–Met conjugate

Various concentrations of HA, Met, the HA–Met conjugate, and a physical mixture of HA + Met were analyzed using high‐performance liquid chromatography (HPLC) on an Agilent 1100 Series system (Agilent Technologies, Santa Clara, CA, USA) equipped with a ultraviolet (UV) detector. Quantification of HA, Met, and the HA–Met conjugate was performed according to the method previously described.^22^

### 2.6 HA–Met conjugate chemical stability in vitro

To evaluate the in vitro release kinetics of Met from the HA–Met conjugate, 1 mL of HA–Met conjugate gel (HA 10 mg, Met 40 mg) or a physical mixture of HA (10 mg) and Met (40 mg) was placed into a dialysis tube with a 7000 Da molecular weight cutoff (Thermo Fisher Scientific, Pittsburgh, PA, USA). The dialysis tube was submerged in 14 mL of phosphate‐buffered saline (PBS) and incubated in a 37°C water bath. The 1 mL sample volume was selected to simulate the clinical dose typically injected into the human knee, and the 14 mL PBS volume approximated the synovial fluid volume of a human knee joint. At each time point, 0.2 mL of the external PBS solution was collected and replaced with an equal volume of fresh PBS. Collected samples were stored at −20°C until analysis. To assess the chemical stability of the HA–Met conjugate, the HA–Met conjugate gel was taken out from the dialysis bag at Day 35 and subjected to boiling in a water bath to cleave the amide bonds. The persistence of the conjugate under these harsh conditions suggests high chemical stability, supporting its potential stability under physiological conditions.

For Met quantification, each 0.2 mL sample collected at each time point was thawed, mixed with 0.8 mL of acetonitrile, vortexed for 30 s, and centrifuged at 12,000 rpm for 10 min. The resulting clear supernatant was collected, and Met concentration was determined using HPLC, following the protocol previously described.^27^ Enzymatic degradation of HA–Met conjugate was performed in two different conditions in 37°C PBS with daily introduction of hyaluronidase 200 or 100 U/mL, to mimic severe inflammation conditions.

### 2.7 Animals

All procedures complied with the Animal Research:Reporting of In Vivo Experiments (ARRIVE) guidelines and were approved by the Institutional Animal Care and Use Committee (IACUC) at the University of Pittsburgh (protocol #23022576). Female C57BL/6J mice (8 weeks old; Jackson Laboratory, Bar Harbor, ME) were housed in the University of Pittsburgh Division of Laboratory Animal Resources (DLAR) facility under standard conditions (22°C, 40%–60% humidity, 12‐h light/dark cycle) with ad libitum access to food and water and were acclimated for ≥7 days before experiments. DMM surgery was performed under inhalational anesthesia and aseptic technique; post‐operative care and analgesia followed the approved protocol.

Mice were randomly assigned to experimental groups prior to any intervention (coin‐toss method). Investigators were not blinded during surgical procedures or injections for logistical reasons; however, blinding was maintained during data collection and outcome assessment, including HPLC quantification and histopathology (Osteoarthritis Research Society International (OARSI) scoring).

Only female mice were used, as pre‐specified, to reduce non‐biological variability in this early efficacy study and to maintain continuity with our laboratory's established PTOA baselines. Female cohorts allow more stable post‐injury group housing with reduced aggression and exhibit tighter body‐weight distributions, helping to standardize post‐injury joint loading—a determinant of PTOA severity—thereby improving statistical power. Estrous staging was not performed; randomization and scheduling distributed procedures across cycle phases, averaging cycle‐related effects at the group level. Future studies will include both sexes to support clinical translation. Another important reason for using female mice in this study was based on 2022 National Health Interview Survey (NHIS) data, which indicated women (21.5%) are more likely than men (16.1%) to have arthritis. Thus, we decided to study the effect of HA–Met conjugate on female OA mice.

### 2.8 Animal allocation

In this study, female C57BL/6J mice (8 weeks old; Jackson Laboratory, Bar Harbor, ME) were used across three experimental settings. For the evaluation of intra‐articular delivery, 64 mice received HA–Met conjugate injections into the knee joint (16 mice at each of four time points). To compare Met alone with the HA + Met mixture, 20 mice were divided into two groups and examined across five time points (two mice per group per time point). Finally, 90 mice were assigned to the OA model study, which included six groups evaluated at three time points (five mice per group per time point). Altogether, these experiments involved 174 mice.

### 2.9 Metformin retention following intra‐articular HA–Met, Met, and HA + Met mixture injections

Mice (16 per group at each of the four time points) received from one to four weekly intra‐articular injections of 10 μL HA–Met conjugate hydrogel (containing 100 μg of hyaluronic acid conjugated with 400 μg of Met per dose) into the knee joint under inhalation anesthesia and aseptic conditions. Knee joint tissues (87.6 ± 5.9 mg/sample), comprising synovium, intra‐articular ligaments, menisci, articular cartilage, subchondral bone up to the growth plate, collateral ligaments, patella, patellar tendon, and synovial membrane, were harvested 1 week after each injection (Weeks 1–4).

Mice (*n* = 2 per group at each of the five time points—1H, 6H, 1D, 2D, and 3D) received one intra‐articular injection of 10 μL of Met solution (containing 400 μg of Met per dose) or HA + Met mixture (containing 100 μg of hyaluronic acid mixed with 400 μg of Met per dose) into the knee joint under inhalation anesthesia and aseptic conditions. Knee joint tissues (75.36 ± 5.96 mg/sample), comprising synovium, intra‐articular ligaments, menisci, articular cartilage, subchondral bone up to the growth plate, collateral ligaments, patella, patellar tendon, and synovial membrane, were harvested. Tissues were homogenized in 0.2 mL PBS, mixed with 0.8 mL acetonitrile, vortexed (1 min), and centrifuged (13,000 rpm, 15 min). Supernatants were collected for Met quantification via HPLC.^27^

### 2.10 Destabilization of medial meniscus‐induced PTOA model

We used an established DMM mouse knee PTOA model^28^ to assess the efficacy of HA–Met gel. A total of 90 eight‐week‐old female C57BL/6J mice were obtained from Jackson Laboratory (Bar Harbor, ME, USA). Under sterile conditions, the right knee area was shaved using depilatory lotion and gauze, then disinfected with 70% ethanol and iodine. A 5 mm longitudinal incision was made from the medial edge of the distal patella to the medial tibial plateau to expose the medial meniscus–tibial ligament. The joint capsule, just medial to the patellar tendon, was incised with a No. 15 blade to open the joint cavity. The fat pad over the intercondylar area was bluntly dissected to visualize the medial meniscus–tibial ligament, which was transected using a No. 11 blade. The capsule and skin were closed with 7–0 Vicryl®. The left knee remained intact.

Following surgery, mice were randomly divided into six groups (*n* = 15/group). Sham (Group 1) received exposure without ligament transection. DMM (Group 2) underwent transection but no treatment. Starting 1 week post‐surgery, Groups 3–6 received weekly intra‐articular injections (10 μL): saline (Sal) (Group 3), HA (10 mg/mL; Group 4), Met (40 mg/mL; Group 5), or HA–Met conjugate (10 mg HA with 40 mg Met/mL; Group 6). At 4, 8, and 12 weeks post‐surgery, five mice from each group were sacrificed for joint tissue analysis.

### 2.11 Histological preparation and staining methods

Mouse knee joint tissues were fixed in 4% formalin for 7 days, then decalcified in 2 mL of Formical‐4 Decalcifier (StatLab) with daily shaking at room temperature. The decalcifying solution was changed every 2 days. Histological services were obtained from HistoWiz, Inc. (Long Island City, NY). Samples were sectioned to a thickness of 5 μm. The area of interest was designated as the medial part of the tibial plateau and femoral condyle in the frontal plane. Every 10th slide was stained with hematoxylin and eosin (H&E), every 11th slide with Safranin O and Fast Green (SFO&FG), and every 12th slide with Masson's trichrome (MT).

### 2.12 Hematoxylin and eosin staining

H&E staining was performed according to a published protocol.^29^ Tissue sections were deparaffinized and rehydrated by passing them through a graded series of alcohol solutions in water. Hematoxylin was applied to stain cell nuclei blue or violet. This was followed by washing, differentiation using an acid‐alcohol solution, and subsequent bluing in an alkaline medium to enhance nuclear contrast. Eosin was then used to stain the cytoplasm and ECM pink. Lastly, the slides were dehydrated using graded alcohols, cleared with xylene, and cover‐slipped for microscopic analysis.

### 2.13 Safranin O and Fast Green staining

SFO&FG staining was performed following a published protocol.^30^ Tissue sections were deparaffinized using xylene and subsequently rehydrated in water. FG was applied to stain collagen and non‐cartilaginous tissues green, followed by a rinsing step. SFO was then used as a counterstain to color cartilage red. Finally, the stained sections were dehydrated, cleared, and mounted for microscopic evaluation.

### 2.14 Masson's trichrome staining

MT staining was performed following a published protocol.^30^ Tissue sections were dewaxed and rehydrated in water. Hematoxylin was used to stain nuclei dark purple, followed by Biebrich Acid Fuchsin to color the cytoplasm and muscle fibers red. The sections were then treated with phosphomolybdic acid and stained with aniline blue to visualize collagen. Finally, the slides were dehydrated, cleared, and mounted for microscopic examination.

### 2.15 OARSI scoring analysis

OARSI scoring was performed on SFO/FG‐stained sections (two slides per sample; six slides from three mice per group; *n* = 3) at 4, 8, and 12 weeks post‐surgery. Three independent, blinded investigators evaluated tibial plateau and femoral condyle regions separately using the 0–6 OARSI scale: 0 = normal; 0.5 = loss of SFO without structural change; 1 = small fibrillations without cartilage loss; 2 = vertical superficial clefts; 3 = vertical clefts/erosion <25% of surface; 4 = 25%–50% of surface; 5 = 50%–75% of surface; 6 = >75% of surface. Scores for tibial and femoral compartments were summed to yield the final OARSI score.^31^ All recommended scoring components—cartilage structure, proteoglycan loss, cellularity, and tidemark integrity—were included. For each joint, three non‐overlapping sections were analyzed, and raw scoring data are presented in Table S1.

### 2.16 Statistical analysis

Statistical analyses were performed in Python (v3.11) using SciPy and statsmodels. Normality of continuous outcomes was assessed using the Shapiro–Wilk test. For intra‐articular Met concentrations, model assumptions were met; therefore, a one‐way analysis of variance (ANOVA) across time (Weeks 1–4) followed by Tukey's Honestly Significant Difference (HSD) post hoc test was used. OARSI scores were summarized as medians with interquartile ranges at 4, 8, and 12 weeks. Because OARSI scores are ordinal and non‐normally distributed, non‐parametric methods were applied. At each time point, group differences among the six treatments (Sham, DMM, saline, HA, Met, and HA–Met) were evaluated using a Kruskal–Wallis test; if significant, pre‐specified Mann–Whitney *U* tests compared HA–Met to DMM, HA, and Met. To control multiplicity while maintaining power, Benjamini–Hochberg false discovery rate (FDR) correction was applied to within‐time point pairwise comparisons (*q* = 0.05), and significance was set at FDR‐adjusted *p* < 0.05. We considered multi‐factor models across time; however, because histopathology outcomes are ordinal and cohorts were independent at each time point (cross‐sectional design), two‐way ANOVA was not appropriate, and per‐time point non‐parametric comparisons were used.

## 3 RESULTS

### 3.1 Fourier‐transform infrared spectroscopy of HA–Met conjugate gel

**FIGURE 1:**
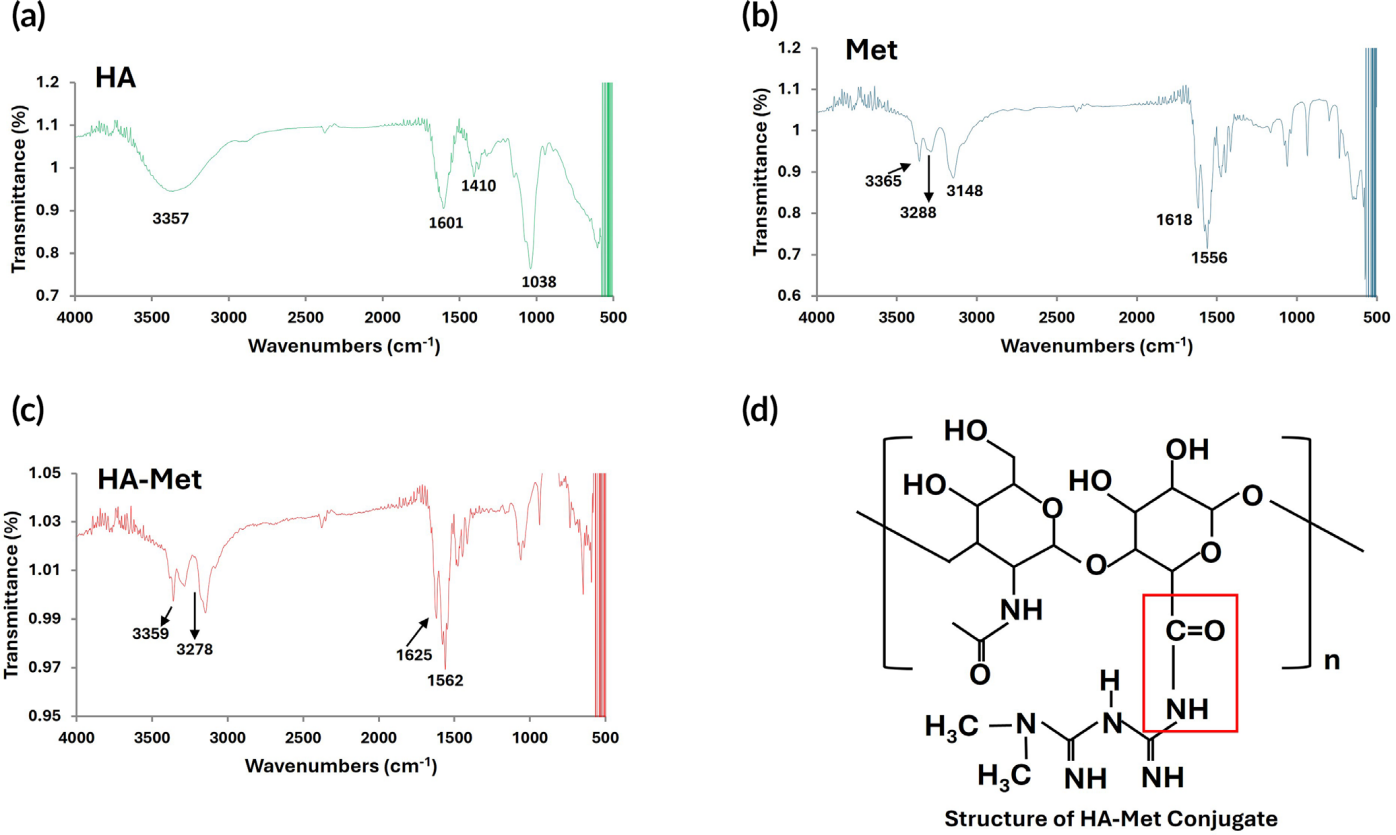
Verification of hyaluronic acid–metformin (HA–Met) conjugation by Fourier‐transform infrared (FT‐IR) spectroscopy. FT‐IR analysis confirmed successful conjugation of Met to HA. (a) FT‐IR spectrum of unmodified HA showing a broad O–H stretching band around ~3400 cm^−1^ and a carboxyl C=O stretching band near ~1600 cm^−1^. (b) FT‐IR spectrum of pure Met displaying strong N–H stretching (~3300 cm^−1^), C=N stretching (~1618 cm^−1^), and N–H bending (~1550 cm^−1^). (c) FT‐IR spectrum of the HA–Met conjugate showing characteristic absorption bands from both HA and Met, including a new peak near ~1625 cm^−1^ corresponding to amide‐bond formation (C=O stretch) and reduced intensity of Met's primary amine (N–H) bands, indicating successful conjugation. (d) Chemical structure of the HA–Met conjugate illustrating the amide‐bond formation between HA and Met (red box).

The conjugation of HA and Met was validated by FT‐IR spectroscopy (Figure 1). The FT‐IR spectrum of HA showed a broad absorption band at 3357 cm^−1^ related to the intra‐ and intermolecular hydrogen bond between the stretching vibration of the OH group and the stretching vibration of the NH group (Figure 1a). The absorption bands at 1601 and 1410 cm^−1^ belonged to the symmetric and asymmetric vibrations of the COO‐ group, respectively (Figure 1a). A strong absorption band at 1038 cm^−1^ corresponded to the hemiacetylic C–O–C of the saccharide unit (Figure 1a). The FT‐IR spectrum of Met showed that the N–H primary asymmetric and symmetric stretching vibrations had broad absorption bands at 3365 and 3288 cm^−1^, respectively (Figure 1b). The N–H in‐plane deformation appeared as a strong band at 1556 cm^−1^ (Figure 1b). In the FT‐IR spectrum of the HA–Met conjugate, a peak at 1625 cm^−1^ corresponded to the amide bond of O=C–N between the carboxylic group of HA and the primary amine group of Met (Figure 1c). Furthermore, the peak at 1556 cm^−1^ involving the N–H bond in the Met spectrum shifted to 1562 cm^−1^ in the spectrum of the HA–Met conjugate (Figure 1c), indicating the formation of an amide bond between the primary amine of Met and the carboxylic acid group of HA (red box Figure 1d).

### 3.2 NMR spectroscopy of HA–Met conjugate

**FIGURE 2:**
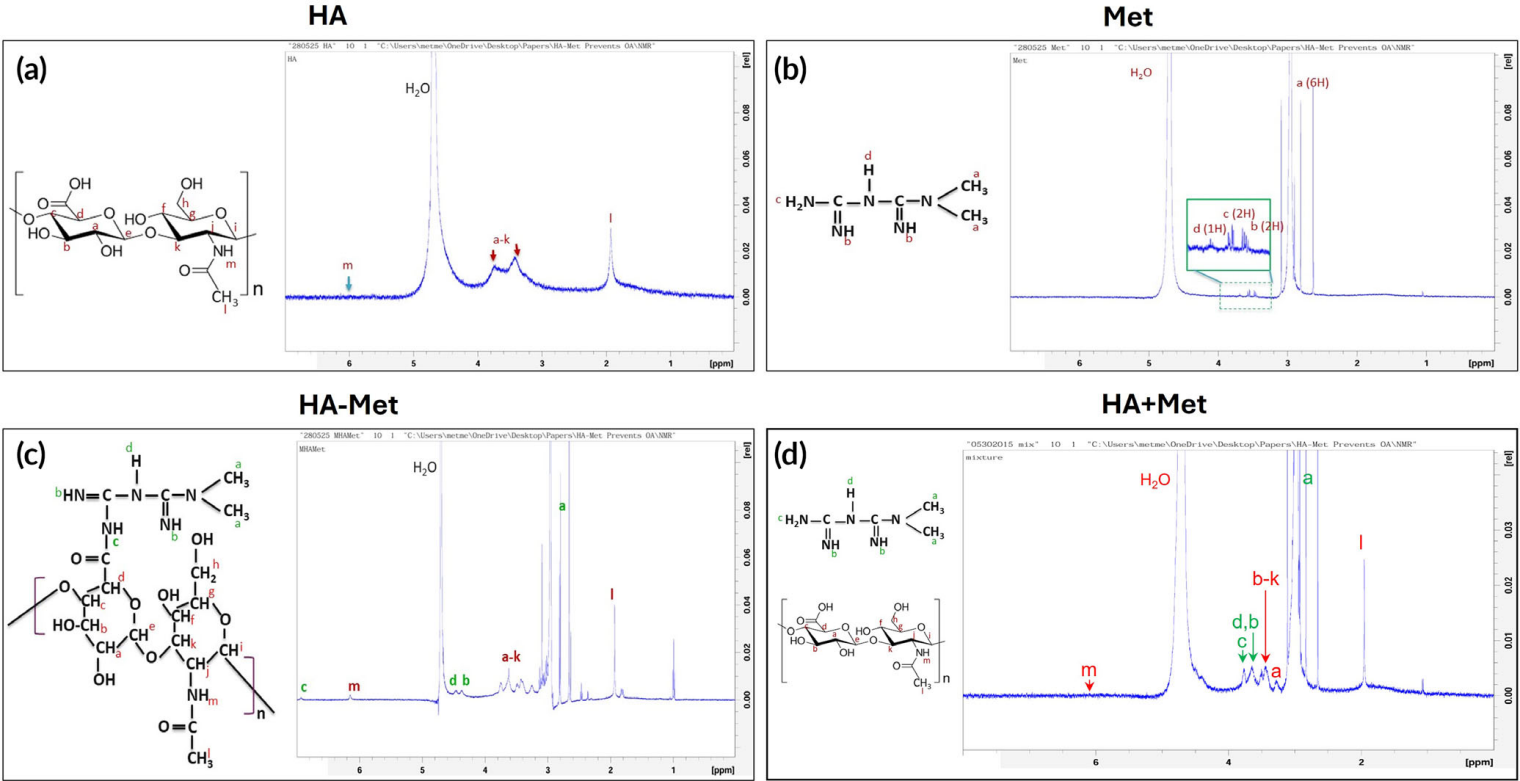
Proton nuclear magnetic resonance spectra confirming the conjugation of metformin (Met) to hyaluronic acid (HA). (a) Spectrum of HA showing *N*‐acetyl methyl protons at 1.99 ppm (red “l”), sugar ring protons from 3.2 to 3.6 ppm, anomeric protons from 3.65 to 4.3 ppm (red “a–k”), weak amide signals at 5.6–6.0 ppm (red “m”), and a solvent peak (HDO) at 4.70–4.90 ppm. (b) Spectrum of Met showing methyl protons in –N(CH_3_)_2_ at 2.8–3.2 ppm (red “a”), –NH‐bound protons at 3.4–3.8 ppm (red “b–d”), and a solvent peak at 4.70–4.90 ppm. Peaks in the green box are magnified from the dashed area. (c) Spectrum of HA–Met conjugate showing HA methyl protons at 1.9 ppm (red “l”). The methyl protons of Met appeared at 2.9 ppm (green “a”), while the sugar ring and anomeric protons of HA were observed at 3.2–3.8 ppm (red “a–k”). The –NH protons of Met shifted to 4.2–4.6 ppm (green “b, d”), and the solvent peak appeared at 4.70–4.90 ppm. Notably, new peaks at 6.10 ppm (HA amide, red “m”) and 6.9 ppm (conjugation‐derived amide, green “c”) confirm successful HA–Met coupling. (d) Spectrum of the simple mixture of HA + Met presented by overlay of HA and Met spectrums without any additional peaks supporting chemical bonding between HA and Met.

The proton NMR spectrum of HA (Figure 2a) showed a distinct peak at ~1.90 ppm corresponding to the methyl group of the *N*‐acetyl moiety (labeled “l” in Figure 2a). Signals from 3.20–3.60 ppm were attributed to sugar ring protons, and those from 3.65 to 4.30 ppm to anomeric protons (labeled “a–k”). A broad peak at 4.70–4.90 ppm was assigned to residual partially deuterated water (HDO) from the D_2_O solvent (labeled “H_2_O”). Weak signals between 5.60 and 6.10 ppm were associated with amide protons of HA.

The ^1^H NMR spectrum of Met (Figure 2b) exhibited characteristic methyl proton signals from the –N(CH_3_)_2_ group at 2.8–3.2 ppm (labeled red “a”), and NH‐bound protons at 3.40–3.80 ppm (labeled red “b–d”). The HDO solvent peak was again observed at 4.70–4.90 ppm (labeled “H_2_O”).

In the HA–Met conjugate spectrum (Figure 2c), chemical shifts differed from those in the individual HA and Met spectra, indicating altered proton environments due to conjugation. The *N*‐acetyl methyl signal of HA (~1.90 ppm, red “l”) and the –N(CH_3_)_2_ signal of Met (~2.90 ppm, green “a”) were both retained with slight shifts. The region from 3.20 to 4.60 ppm showed overlapping peaks, contributed by HA sugar ring protons (red “a–k”) and Met NH protons (green “b” and “d”). The residual HDO peak remained at 4.70–4.90 ppm.

Notably, two new downfield peaks appeared at ~6.10 and ~6.90 ppm (Figure 2c). The former likely corresponds to HA amide protons (red “m” in Figure 2c), while the latter represents newly formed amide protons (–O=C–NH–) from the conjugation reaction (green “c” in Figure 2c), confirming the successful linkage of HA and Met.

For comparison, a physical mixture of HA + Met (Figure 2d) produced a simple overlay of the two individual spectra, lacking the spectral shifts and new peaks observed in the HA–Met conjugate, further supporting successful chemical conjugation.

### 3.3 HPLC analysis of HA–Met conjugate gel

**FIGURE 3:**
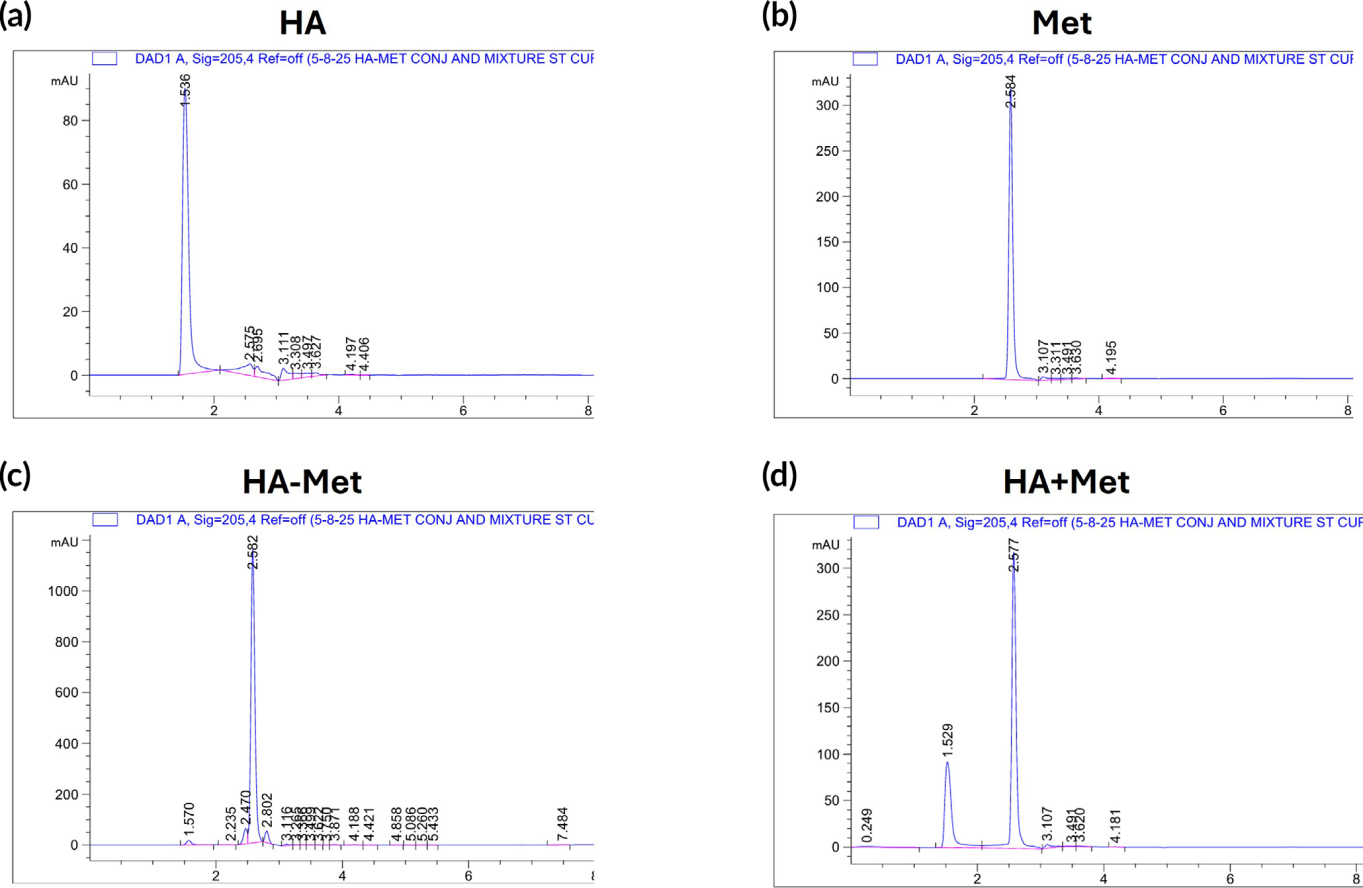
High‐performance liquid chromatography chromatograms demonstrating successful conjugation of metformin (Met) to hyaluronic acid (HA). (a) Chromatogram of unmodified HA showing a distinct peak at retention time (RT) 1.536 min with no signal at the metformin RT (2.6 min). (b) Chromatogram of free metformin displaying a sharp peak at RT 2.6 min and no signal at the HA RT (1.5 min). (c) Chromatogram of the HA–Met conjugate showing a minimal HA peak at ~1.5 min and a strong peak at RT 2.582 min, along with additional peaks at ~2.4 and ~2.8 min, confirming successful conjugation. (d) Chromatogram of the HA + Met simple mixture showing an overlay of the pure HA and pure Met peaks without any additional peaks, indicating the absence of bond formation between HA and Met. mAU= milli‐Absorbance Units.

The chromatogram of pure HA (Figure 3a) showed a major peak at approximately 1.5 min, while Met (Figure 3b) exhibited a peak at around 2.6 min. In the HA–Met conjugate sample (Figure 3c), the free HA peak at a retention time (RT) of 1.5 min disappeared, and new peaks emerged at approximately 2.4 and 2.8 min, corresponding to HA–Met conjugates with different molecular weights (Figure 3c). A physical mixture of HA + Met (Figure 3d) yielded a simple overlay of the individual HA (RT at 1.529 min) and Met (RT at 2.577 min) peaks, without the additional peaks observed in the conjugate sample.

### 3.4 In vitro stability of HA–Met conjugate gel

**FIGURE 4:**
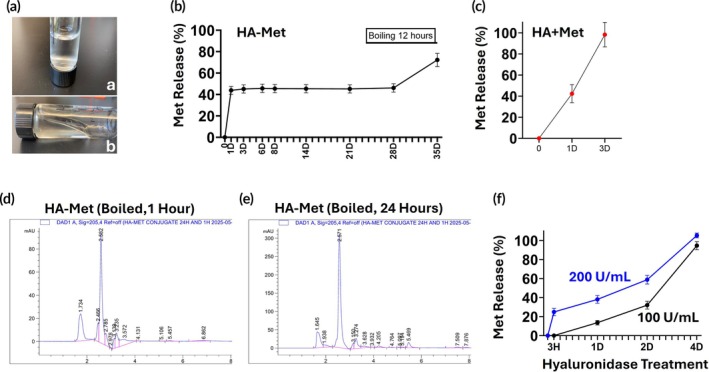
Physical appearance and in vitro stability of hyaluronic acid–metformin (HA–Met) conjugate. (a) HA–Met conjugate appears as a transparent, viscous liquid under static conditions (a), and flows within 40 s when tilted (b). (b) The conjugate remains stable in vitro for at least 28 days. Boiling the sample for 12 h cleaves amide bonds, resulting in the release of additional Met. (c) Met releases from HA + Met mixture within 3 days. (d) After 1 h of boiling, the HA–Met conjugate remains largely intact, with characteristic peaks of conjugated molecules still visible at approximately 2.4 and 2.8 min. (e) Disintegration of the HA–Met conjugate is evident only after 24 h of boiling, marked by the disappearance of conjugated peaks and the emergence of distinct HA and Met peaks. The HA peak is slightly shifted to ~1.6 to 1.7 min, and additional peaks appearing after 3 min are presumed to be degradation products of the conjugate. (f) Enzymatic release of Met from HA–Met conjugate with daily introduction of hyaluronidase 200 and 100 U/mL. Note that the additional release observed between 28 and 35 days corresponds to a separate accelerated stability test in which the conjugate was boiled for 12 h to confirm chemical stability. The 28‐day time course reflects physiological conditions, whereas the extended “boiling” data represent a stress condition included to demonstrate conjugate stability.

The HA–Met gel was prepared by dissolving dried HA–Met conjugate in deionized water, yielding a transparent, viscous solution (Figure 4a; quiescent state, panel a; flow initiated after 40 s, panel b). Stability testing showed that approximately 40% of Met was detected in the gel solution on Day 1 (Figure 4b), corresponding to un‐conjugated Met entrapped within the HA–Met conjugate. This concentration remained constant at 37°C for 28 days, indicating excellent in vitro stability. When the gel was subjected to thermal stress (100°C, 12 h), >72% of Met was released into solution (Figure 4b). By contrast, a physical mixture of HA + Met exhibited rapid release, with nearly complete release observed within 3 days (Figure 4c). These results indicate that the HA–Met conjugate gel is highly stable under physiological conditions, and only prolonged boiling induced partial conjugate degradation with additional Met release (Figure 4b). The observed increase in Met signal beyond 28 days reflects this accelerated hydrolysis experiment and is not part of the 28‐day physiological incubation.

Furthermore, HPLC analysis showed minimal HA in the unheated HA–Met conjugate gel solution (Figure 3c). After boiling at 100°C for 1 h, the HA peak at RT 1.7 min increased (Figure 4d). Further heating for 24 h led to more extensive hydrolysis, evidenced by a twofold increase in HA, the appearance of a Met peak at ~2.6 min, and the disappearance of conjugate‐specific peaks at ~2.4 and ~2.8 min (Figure 4e). Enzymatic degradation of the HA–Met conjugate was assessed by adding hyaluronidase daily at concentrations of 200 or 100 U/mL. The cumulative Met release reached 24.84% and 0% at 3 h, 38.08% and 13.56% at 1 day, 58.82% and 32% at 2 days, and 105.31% and 94.67% at 4 days for the 200 and 100 U/mL groups, respectively (Figure 4f).

### 3.5 In vivo detection of Met following intra‐articular HA–Met, Met, and HA + Met injections

**FIGURE 5:**
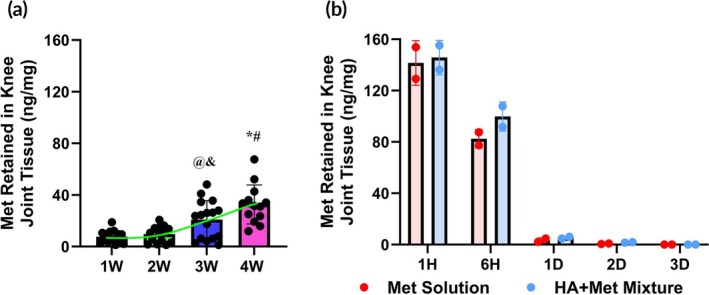
Local retention of metformin (Met) in the mouse knee joint. Weekly injections of hyaluronic acid–metformin (HA–Met) conjugate gel (a) and one time injection of HA + Met mixture or Met solution (b). Mice received four weekly intra‐articular injections of 10 μL HA–Met conjugate gel (containing Met at 40 mg/mL; total Met dose per injection = 400 μg). Knee joint tissues (87.62 ± 5.86 mg/sample) were harvested 1 week after each injection. The amount of Met retained in the knee joint increased progressively: From 7.61 ng/mg tissue after 1 week of the first injection to 9.7, 20.86, and 32.62 ng/mg following the second, third, and fourth injections, respectively (a). @ represents *p* = 0.012 (1W vs. 3W); & represents *p* = 0.039 (2W vs. 3W); * represents *p* = <0.0001 (1W vs. 4W and 2W vs. 4W); # represents *p* = 0.036 (3W vs. 4W). Mice received a single intra‐articular injection of 10 μL of the HA + Met mixture (containing metformin at 40 mg/mL; total Met dose = 400 μg). Knee joint tissues (75.36 ± 5.96 mg per sample) were collected at 1 h, 3 h, and 1–3 days post‐injection. The Mer retention in the knee joint was comparable between the Met solution and HA + Met mixture groups and declined rapidly over time—from 141.49 and 145.74 ng/mg at 1 h, to 82.48 and 99.74 ng/mg at 6 h, 3.45 and 5.43 ng/mg at 1 day, 0.71 and 1.74 ng/mg at 2 days, and 0 and 0.06 ng/mg at 3 days, respectively (b).

After HA–Met conjugate injections, the amount of Met retained in knee joint tissues increased progressively over time, rising from 7.6 ng/mg tissue after the first weekly injection to 9.7, 20.9, and 32.6 ng/mg following the second, third, and fourth injections, respectively (Figure 5a). In contrast, the amount of Met retained in the knee joint after Met solution or HA + Met mixture injections was almost similar at every time point and rapidly decreased after injection from 141.49 and 145.74 ng/mg in 1 h, 82.48 and 99.74 ng/mg in 6 h, 3.45 and 5.43 ng/mg in 1 day, 0.71 and 1.74 ng/mg in 2 days to 0 and 0.06 ng/mg in 3 days (Figure 5b).

To assess changes in local Met levels in the knee joint tissues over time, we performed one‐way ANOVA, which revealed a significant effect of time on tissue drug concentration (*p* < 0.0001). Post hoc Tukey's HSD tests identified several significant pairwise differences: (1) Met levels at 4 weeks were significantly higher than at 1W (*p* < 0.001), 2W (*p* < 0.001), and 3W (*p* = 0.02); (2) levels at 3W were also significantly higher than at 1W (*p* = 0.004) and 2W (*p* = 0.012); (3) no significant difference was observed between 1W and 2W (*p* > 0.05).

These findings confirm that HA–Met gel enables sustained and time‐dependent retention of Met in joint tissues, with peak levels achieved at 4 weeks post‐injection.

### 3.6 HA–Met conjugate gel injections mitigate PTOA development

**FIGURE 6:**
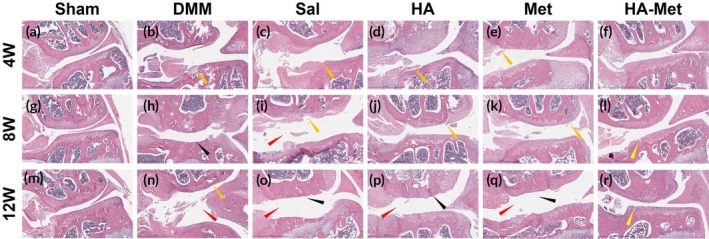
Hyaluronic acid–metformin (HA–Met) gel injections mitigate post‐traumatic osteoarthritis (PTOA) progression in the mouse knee joint. Hematoxylin & eosin (H&E) staining reveals that the Sham group maintains normal cartilage architecture at all time points (a, g, m). In contrast, the destabilization of medial meniscus (DMM) and saline groups exhibit typical osteoarthritic changes, validating the PTOA model (b, h, n and c, i, o, respectively). The HA and Met alone groups also demonstrate PTOA development and progression, though to a lesser extent than the DMM and Saline (Sal) groups, suggesting partial protective effects (d, j, p, and e, k, q, respectively). Notably, HA–Met gel injections preserve or restore cartilage structure, closely resembling normal histology (f, l, and r).

H&E staining indicated that the Sham group had normal cartilage thickness and subchondral bone without pathological changes, normal meniscus condition, and location at all time points (Figure 6a,g,m).

There was a gradual increase in cartilage, meniscus, and subchondral bone osteoarthritic changes in the DMM‐only group at all time points (Figure 6b,h,n) justifying the success of the DMM model. The model also showed articular cartilage roughness and clefts (orange arrow in Figure 6b,n), deep cartilage erosions extending into the subchondral bone (black arrow in Figure 6h), and widening of the joint gap with a change of correlation between joint components (red arrow in Figure 6n).

Saline group (Figure 6c,i,o) showed joint gap widening and deformation (red arrow in Figure 6i,o), cartilage roughness, tears, and clefts (orange arrow in Figure 6c,i) at 4 and 8 weeks. Moreover, at the 12 weeks time point, massive deep to subchondral bone cartilage erosions were detected at both condyles (black arrow in Figure 6o).

In the HA group (Figure 6d,j,p), there were cartilage tears and clefts in both femoral and tibial condyles at the earliest term of the study, almost without progression to the 8‐week time point (orange arrow in Figure 6d,j) and rapid pathological changes progressed to the latest time point, expressed by joint gap widening and deformation (red arrow in Figure 6p), and massive cartilage erosions with subchondral bone pathological changes (black arrow in Figure 6p).

As in the HA group, the Met group (Figure 6e,k,q) showed similar changes in PTOA progression expressed by cartilage fibrillations and tears at the early time points (orange arrow in Figure 6e,k), massive cartilage erosions deep to subchondral bone, with subchondral bone pathological changes at 12 weeks (black arrow in Figure 6q) as well as joint gap widening and deformation (red arrow in Figure 6q). These results show that HA and Met alone are not sufficient to block PTOA development.

In contrast, the HA–Met group (Figure 6f,l,r) displayed normal cartilage thickness, minimal cartilage fibrillations without loss of cartilage at the 4 weeks time point (Figure 6f), and minimal cartilage fibrillations and clefts at 8 and 12 weeks time points (yellow arrow in Figure 6l,r), demonstrating the preventive effects of the HA–Met gel.

**FIGURE 7:**
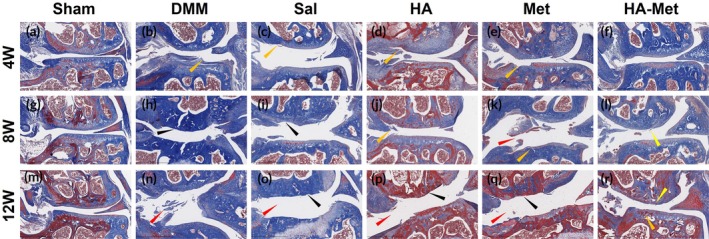
Hyaluronic acid–metformin (HA–Met) gel injections preserve cartilage integrity in the mouse knee joint. Masson's trichrome (MT) staining shows that the Sham group has normal cartilage, subchondral bone, and meniscus condition at all time points with normal collagen expression (a, g, m). The destabilization of medial meniscus (DMM) and saline groups exhibit typical pathological changes of post‐traumatic osteoarthritis (PTOA) (b, h, n and c, i, o, respectively). The HA and Met groups demonstrate almost similar changes indicating PTOA progression (d, j, p and e, k, q, respectively). On the contrary, HA–Met group shows normal joint tissue conditions (f, l, r).

MT staining further confirmed the previous staining results (Figure 7). Normal cartilage, subchondral bone, and meniscus condition were detected in the Sham group at all time points (Figure 7a,g,m). In the DMM and Saline groups at the 4 weeks time point, cartilage fibrillations, roughness, and cartilage clefts were evident (orange arrow in Figure 7b,c, respectively). At the 8 weeks time point, we observed similar changes in both groups described as massive cartilage erosions deep to subchondral bone (black arrow in Figure 7h,i, respectively). At the 12 weeks time point, we observed the deformation and widening of the joint gap (red arrow in Figure 7n,o), and cartilage erosions deep to subchondral bone in the met group (black arrow in Figure 7o).

In HA only and Met only groups at the 4 weeks time point, we observed cartilage fibrillations and clefts (orange arrow in Figure 7d,e respectively) in both condyles, and expressed similar changes at the 8 weeks time points (orange arrow in Figure 7j,k, respectively) as well as joint gap widening in the Met only group (red arrow in Figure 7k). Finally, at the 12 weeks time point, erosions deep to the subchondral bone and joint gap widening were detected (black and red arrows in Figure 7p,q, respectively). These results suggest that HA and Met alone treatments were not able to prevent PTOA development. However, in the HA–Met conjugate gel group at the 4 weeks time point, mostly normal joint components condition was detected (Figure 7f). At the 8 and 12 weeks time points, cartilage fibrillations and roughness were detected (yellow arrow in Figure 7l,r) with some cartilage clefts in both condyles at the 12 weeks time point (orange arrow in Figure 7r).

**FIGURE 8:**
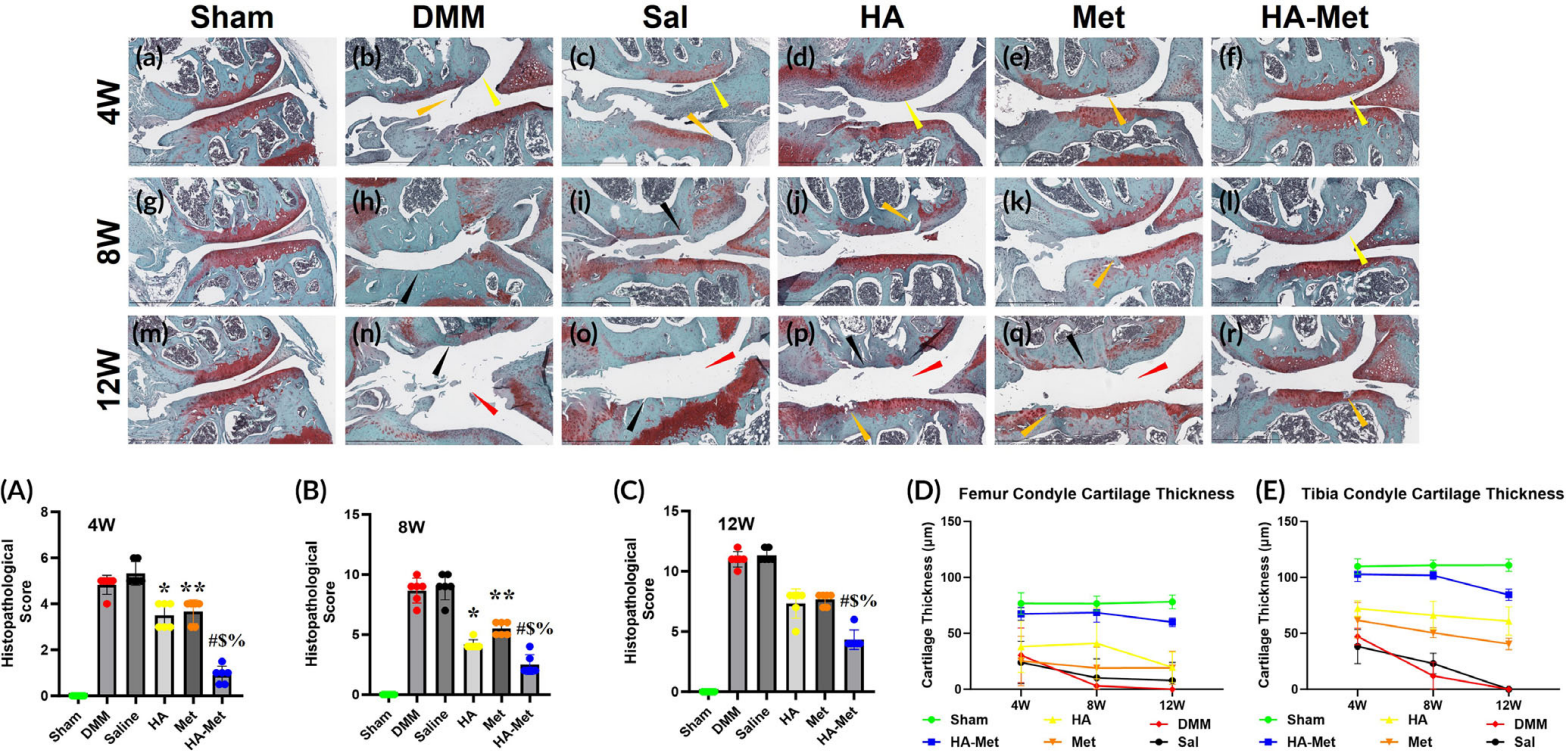
Hyaluronic acid–metformin (HA–Met) gel injections attenuate post‐traumatic osteoarthritis (PTOA) progression in the mouse knee joint. Safranin O and Fast Green (SFO&FG) staining reveals that the Sham group maintains normal cartilage and meniscus condition with intensive proteoglycans expression and normal subchondral bone condition (a, g, m). The destabilization of medial meniscus (DMM) and saline groups show pathological changes indicating PTOA progression (b, h, n and c, i, o, respectively), whereas the HA and Met groups show less rapid progression of pathological changes compared to DMM and Saline (d, j, p and e, k, q, respectively). But the HA–Met group restores normal cartilage structure (f, l, r). The pathological changes were assessed using OARSI scoring at 4 weeks (A), 8 weeks (B), and 12 weeks (C). Statistical analysis of SFO&FG staining revealed significant group differences at multiple time points (*n* = 6 per group). At 4 weeks, HA versus DMM: *p* = 0.0005 (*); Met versus DMM: *p* = 0.0005 (**); HA–Met versus HA: *p* = 0.0057 (#); HA–Met versus Met: *p* = 0.0057 ($); and HA–Met versus DMM: *p* = 0.0004 (%). At 8 weeks, HA versus DMM: *p* = 0.0005 (*); Met versus DMM: *p* = 0.0011 (**); HA–Met versus HA: *p* = 0.0089 (#); HA–Met versus Met: *p* = 0.0057 ($); and HA–Met versus DMM: *p* = 0.0005 (%). At 12 weeks, HA–Met versus HA: *p* = 0.0065 (#); HA–Met versus Met: *p* = 0.0127 ($); and HA–Met +versus DMM: *p* = 0.0005 (%). Femur (D) and tibia (E) condyles cartilage thickness semi‐quantification shows dramatic decrease in DMM, Sal, HA, and Met groups at all time points, and slight decrease of cartilage thickness in the HA–Met group at 12 weeks compared to the Sham group.

SFO&FG staining confirmed the H&E staining results (Figure 8). In the Sham group (Figure 8a,g,m), we observed normal cartilage and meniscus condition with intensive proteoglycans expression at all time points. In the DMM (Figure 8b,h,n) and Saline groups (Figure 8c,i,o) at the 4 weeks time point, we observed similar changes characterized by cartilage roughness and proteoglycans expression lost (yellow arrow in Figure 8b,c) with cartilage clefts in both condyles (orange arrow in Figure 8b,c). At the 8 and 12 weeks time points (Figure 8h,i,n,o respectively), massive cartilage erosions and fragmentation were observed in both condyles with pathological changes in subchondral bone (black arrow in Figure 8h,i,n,o) as well as widening of the joint gap with change of correlation between joint components at the 12 weeks time point (red arrow in Figure 8n,o). The changes described above are additional proof of PTOA surgical model efficacy, providing evidence of progressive PTOA.

In the HA group at the 4 weeks time point (Figure 8d), we detected proteoglycan expression loss without structural changes in femur condyle cartilage (yellow arrow in Figure 8d) and mostly normal proteoglycans expression in tibial condyle. At the 8 weeks time point, we detected proteoglycans loss with cartilage clefts in femur condyle (orange arrow in Figure 8j) and mostly normal proteoglycans expression in tibial condyle. At the 12 weeks time point (Figure 8p), we detected massive cartilage erosions in femoral condyle (black arrow in Figure 8p) with a deformed and widened joint gap (red arrow in Figure 8p) and proteoglycans lost with cartilage clefts in tibial condyle (orange arrow in Figure 8p).

In the Met group (Figure 8e,k,q), we noted cartilage proteoglycans loss combined with cartilage clefts and fibrillations in both condyles at all time points (orange arrow in Figure 8e,k,q) and at 12 weeks (q) cartilage erosions of femoral condyle (black arrow in Figure 8q) and joint gap widening and deformation (red arrow in Figure 8q). Histopathological changes in HA and Met groups are very similar. These data indicate that treatments with HA or Met alone are not effective in preventing PTOA progression. In contrast, after injections with HA–Met gel (Figure 8f,l,r), the knee joints showed mostly normal proteoglycans expression with mild superficial proteoglycans loss at 4 and 8 weeks (yellow arrow in Figure 8f,l), and some minor cartilage clefts at 12 weeks (orange arrow in Figure 8r), suggesting the effectiveness of HA–Met gel in preventing PTOA progression.

The pathological changes of the tibial plateau and femoral condyle medial part cartilage and subchondral bone were assessed using OARSI scoring, and the scores agree with the above results (Figure 8A‐C ). Normal joint condition with 0 pathological scores was observed in the Sham group at all time points of the study, and they represent values typical for healthy joint cartilage and subchondral bone condition. However, in the DMM group, the OARSI scores were 4.8, 8.7, and 11.0, and in the saline group, they were 5.3, 9.0, and 11.3 at 4 (Figure 8A), 8 (Figure 8B), and 12 (Figure 8C) weeks, respectively, indicating progressive pathological changes characteristic of PTOA.

The scores for the HA group were 3.5, 4.2, and 7.3, and for the Met group, they were 3.7, 5.5, and 7.7 at 4, 8, and 12 weeks' time points, respectively (Figure 8A‐C). These scores are slightly lower than those of the DMM and Saline groups, indicating at least some protective effects of each one when administered separately. On the contrary, the HA–Met group showed a gradual increase in pathological scores, starting from a low 0.91 at the 4 weeks' time point, 2.5 at the 8 weeks' time point, and 4.3 at the 12 weeks' time point, indicating the effectiveness of HA–Met gel in mitigating PTOA progression (Figure 8A‐C).

OARSI scores were compared between treatment groups at 4, 8, and 12 weeks using the Mann–Whitney U test. At all three time points, HA–Met treatment significantly reduced histopathological OARSI scores compared to the DMM group (FDR‐adjusted *p* < 0.001). Compared to HA or Met monotherapy, HA–Met treatment also showed significantly improved outcomes at 4 weeks (*p* = 0.0057 for both comparisons), 8 weeks (*p* = 0.0089 and *p* = 0.0057), and 12 weeks (*p* = 0.0065 and *p* = 0.0127). Both HA and Met groups exhibited significantly lower scores than DMM at 4 and 8 weeks (all *p* < 0.001), but not at 12 weeks (HA vs. DMM: *p* = 0.0547; Met vs. DMM: *p* = 0.0570).

Additional semi‐quantification of femur condyle cartilage thickness (Figure 8D) showed dramatic thickness decrease at the 4‐week time point in DMM—30.38 μm, Sal—24.01 μm, HA—38.15 μm, and Met 25.34 μm. In contrast, the HA–Met conjugate group showed only a slight decrease in cartilage thickness (67.74 μm) compared to the sham group (76.69 μm). At the 8‐week time point, Sham, HA–Met, and HA groups' cartilage thickness changed insignificantly and was 76.60, 68.52, and 41.07 μm, respectively, whereas in Met, Sal, and DMM groups we observed a decrease in cartilage thickness to 19.04, 10.31, and 3.09 μm, respectively. Finally, at the 12‐week time point, the cartilage thickness in the sham group remained nearly unchanged at 78.19 μm. The HA–Met group showed a moderate decrease to 60.0 μm, while the Met group exhibited little change, with a thickness of 19.19 μm. In contrast, the HA group showed a reduction to 19.7 μm, and the saline and DMM groups displayed severe cartilage loss, with thicknesses of 7.97 and 0 μm, respectively (Figure 8D).

Semi‐quantitative analysis of tibial condyle cartilage thickness (Figure 8E) revealed a trend similar to that observed in the femoral condyle. From 4 to 12 weeks, cartilage thickness remained stable in the sham group (109.75, 110.81, and 110.96 μm) but progressively decreased in the treatment groups: HA–Met conjugate (102.78 → 84.43 μm), HA (72.18 → 60.91 μm), and Met (61.71 → 40.54 μm). Severe cartilage loss was observed in the DMM (47.20 → 0 μm) and saline (38.26 → 0 μm) groups.

## 4 DISCUSSION

In this study, we developed an injectable HA–Met conjugate gel and demonstrated its potential as a localized intra‐articular delivery platform with prolonged retention and stability of Met in joint tissues. Although PTOA was used as the proof‐of‐concept indication, the broader significance lies in the drug‐delivery design: covalent coupling of Met to HA enables targeted intra‐articular exposure while limiting systemic distribution.

HA‐based delivery platforms are widely used in musculoskeletal applications owing to their biocompatibility, viscoelasticity, and ability to localize therapeutics within joint compartments.^32^ In our approach, HA contributes mechanical support and lubrication, whereas Met establishes an anti‐inflammatory, antioxidant microenvironment. Importantly, covalent conjugation distinguishes this system from prior encapsulation‐based strategies, which can permit rapid drug escape and variable release profiles. By addressing key limitations of intra‐articular Met—short half‐life, rapid synovial clearance, and the need for frequent redosing—this conjugate targets a well‐defined translational gap.

Multiple orthogonal analytical methods confirmed conjugation and informed release behavior. FT‐IR, ^1^H NMR, and HPLC collectively supported amide‐bond formation between HA and Met, consistent with stable covalent linkage. In vitro studies showed that the initial 24‐h burst primarily reflects unbound drug, whereas the conjugated fraction remained intact under physiological incubation for at least 28 days. The apparent “late” release seen between Days 28 and 35 corresponded to a separate stress test in which samples were boiled for 12 h to accelerate hydrolysis; these data were included solely to confirm the chemical stability of the conjugate and are not part of the physiological time course. In vivo, Met was detectable in joint tissues for up to 7 days after a single injection of the HA–Met conjugate gel, and levels increased with weekly administration, indicating progressive local retention. For analytical detection in biological matrices, we used complementary HPLC methods: the assay of Huttunen et al. enhanced Met resolution, while the method of Boltia et al. enabled simultaneous HA/Met detection.^22^, ^27^

The weekly dosing used in mice was selected to maintain continuous therapeutic exposure in a small‐animal model with rapid metabolism and joint clearance. Using standard physiological time‐scaling principles, a weekly regimen in mice approximates substantially longer intervals in humans (on the order of 1–2 months), and should therefore be interpreted as an accelerated proof‐of‐concept design rather than a proposed clinical schedule. Ongoing pharmacokinetic and residence‐time studies in larger animals are expected to define clinically relevant dosing frequencies.

Compared with conventional intra‐articular injections or systemic pharmacologic approaches, this HA–Met conjugate offers several potential advantages: (i) localization reduces the risk of systemic adverse effects; (ii) prolonged local retention may decrease injection frequency compared with free drug; and (iii) the combination of HA‐mediated joint support with Met's anti‐inflammatory activity may provide dual‐action protection. Alternative sustained‐delivery strategies—such as liposomal corticosteroids and poly(lactic‐co‐glycolic acid) (PLGA) microspheres—are under investigation,^33^, ^34^ but relatively few systems combine long‐term intra‐articular residence, intrinsic bioactivity, and simple administration as effectively as an HA‐conjugate. Likewise, Met‐loaded HA‐liposome hydrogels have shown benefit in disc degeneration,^35^ yet they rely on physical encapsulation rather than covalent linkage, which can compromise release stability and reproducibility.

This work has limitations that inform future studies. First, while we compared conjugated versus non‐conjugated (HA + Met mixture) formulations in vitro and found rapid release/clearance of free Met relative to the conjugate, we did not include an HA + Met mixture control in vivo; future efficacy studies will incorporate this control to directly test attribution of benefit to conjugation. Second, we did not assess mechanical/rheological properties of the gels; these material characterizations will be important for optimization and translation. Third, we did not evaluate the bioactivity of Met after extended incubation; follow‐up studies will pair long‐term release with functional readouts. Additional considerations include the use of female mice without estrous staging (mitigated by randomization across cycle phases), the inherent constraints of the DMM surgical model, and the need for expanded mechanistic analyses (e.g., cytokine signaling, oxidative stress, and ECM remodeling).

Looking forward, key translational steps include dose‐ranging and durability studies to define exposure and residence time, optimization of dosing frequency, confirmation of efficacy in both sexes and relevant hormonal states (e.g., post‐menopausal), and evaluation in larger animal models. Manufacturing objectives include a sterile, single‐use formulation with validated stability/sterilization parameters, followed by good laboratory practice (GLP) toxicology to support regulatory submissions. Collectively, these efforts will determine whether the HA–Met conjugate gel can offer a practical, scalable approach to delay or prevent PTOA progression in high‐risk patients following joint injury.

## AUTHOR CONTRIBUTIONS

**Vasyl Pastukh**: Writing original draft, Investigation, data curation, formal analysis, validation. **Jianying Zhang**: Methodology, data curation, validation. **Soichi Hattori**: Methodology, data curation, validation. **Susheng Tan**: Methodology. **Satyaj Bhargava**: Methodology, investigation. **Derek Maloney**: Methodology, investigation. **MaCalus V. Hogan**: Writing review and editing. **James H‐C. Wang**: Conceptualization, project administration, methodology, validation, resources, writing—review and editing, supervision.

## FUNDING INFORMATION

This work was supported in part by a Department of Defense/Medical Technology Enterprise Consortium (DOD/MTEC) award W81XWH2290016 and DOD HT9425‐23‐1‐0617.

## CONFLICT OF INTEREST STATEMENT

The authors declare no conflicts of interest.

## Supporting information


**Figure S1.** Schematic illustration of the chemical conjugation of hyaluronic acid (HA) with metformin (Met). The carboxyl groups on HA were activated using EDC/NHS chemistry to form amide bonds with the guanidine group of Met, resulting in the HA–Met conjugate. This reaction enables covalent linkage of Met to the HA backbone, facilitating controlled release and sustained bioactivity in joint tissues.

**Table S1.** Raw OARSI scores for all animals at 4, 8, and 12 weeks.

## Data Availability

The data that support the findings of this study are available from the corresponding author upon reasonable request.
