# Supplementary figures and images for: Injectable hyaluronic acid–metformin conjugate gel for sustained intra‐articular delivery and prevention of post‐traumatic osteoarthritis
Source: Bioeng Transl Med. 2025 Dec 25;11(1):e70100. doi: 10.1002/btm2.70100 (PMC12821215; doi:10.1002/btm2.70100)

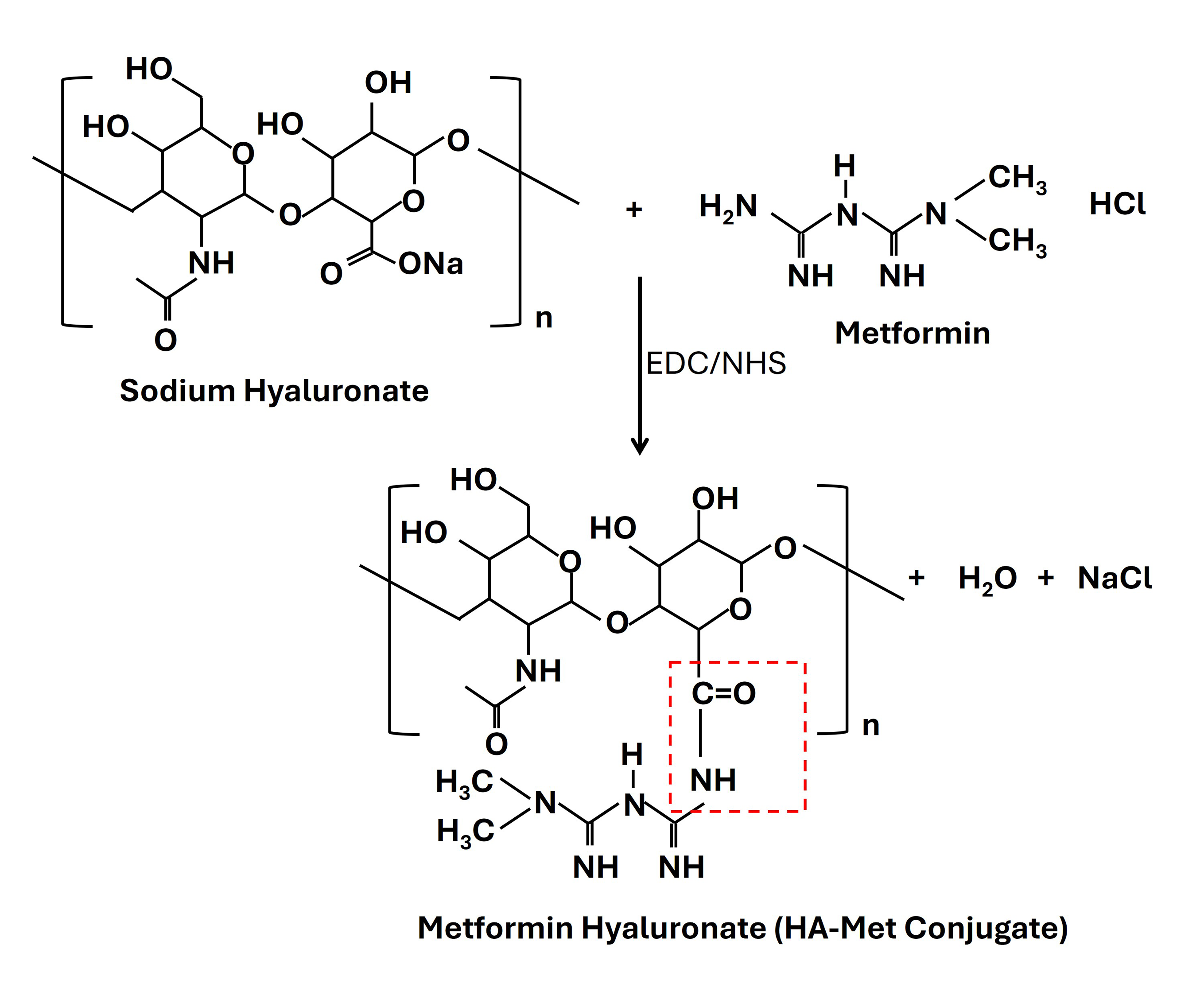

Supplement: Supplementary file 1 — Figure S1. Schematic illustration of the chemical conjugation of hyaluronic acid (HA) with metformin (Met). The carboxyl groups on HA were activated using EDC/NHS chemistry to form amide bonds with the guanidine group of Met, resulting in the HA–Met conjugate. This reaction enables covalent linkage of Met to the HA backbone, facilitating controlled release and sustained bioactivity in joint tissues. [file BTM2-11-e70100-s002.tif]
