# Supplementary material for: Injectable hyaluronic acid–metformin conjugate gel for sustained intra‐articular delivery and prevention of post‐traumatic osteoarthritis
Source: Bioeng Transl Med. 2025 Dec 25;11(1):e70100. doi: 10.1002/btm2.70100 (PMC12821215; doi:10.1002/btm2.70100)
Supplement: Supplementary file 2 — Table S1. Raw OARSI scores for all animals at 4, 8, and 12 weeks. [file BTM2-11-e70100-s001.docx]

| **Table S1. Raw OARSI Scores for All Animals at 4, 8, and 12 Weeks** | | | | | | |
| --- | --- | --- | --- | --- | --- | --- |
| **Time** | **Sham** | **DMM** | **Saline** | **HA** | **Met** | **HA–Met** |
| **4 Weeks (Fig. 8a)** | 0  0  0  0  0  0 | 5  5  5  5  4  5 | 5  5  6  6  5  5 | 3  4  4  3  3  4 | 4  4  4  3  3  4 | 0.5  1  1.5  1  1  0.5 |
| **8 Weeks**  **(Fig. 8b)** | 0  0  0  0  0  0 | 8  10  7  9  9  9 | 9  10  7  9  9  10 | 4  4  5  4  4  4 | 6  5  5  6  6  5 | 2  2  3  2  4  2 |
| **12 Weeks**  **(Fig. 8c)** | 0  0  0  0  0  0 | 11  12  10  11  11  11 | 11  12  12  11  11  11 | 5  8  7  8  8  8 | 7  7  8  8  8  8 | 4  4  4  4  6  4 |
